# Sex differences in the strategies of haptic object memory in early adulthood

**DOI:** 10.1186/s13293-026-00880-2

**Published:** 2026-03-24

**Authors:** Lea Fröscher, Matthias W. Riepe

**Affiliations:** Geriatric Psychiatry Section, Clinic for Psychiatry and Psychotherapy II of the University of Ulm at Günzburg District Hospital, Günzburg, Germany

**Keywords:** sex differences, haptic object memory, touch, cognitive strategies, verbal and spatial processing

## Abstract

Everyday life inevitably involves touch. Encoding, recalling, and recognizing objects by touch requires haptic object memory, which is thought to rely on both verbal and visual capabilities. This raises the question of whether performance or strategies used to solve haptic memory tasks are sex-dependent. We assessed 45 healthy young adults (27 females, 18 males; aged 18–36 years) with repeated measurements. Participants were asked to encode ten everyday objects while blindfolded and allowed to explore them by touch. After a five-minute delay, participants were asked to recall as many objects as possible and subsequently to recognize the objects from a set of five conceptually identical but perceptually different alternatives with their eyes open. Regardless of whether participants correctly identified the object, they were then allowed to touch it. Performance did not differ between women and men in all memory phases. However, men appeared to rely less on verbal memory and instead used mental rotation as a compensatory strategy during encoding. Women who in this sample showed better verbal memory performance, did not require such backup strategies for encoding. During the recognition task, the results suggest that men may have relied on mental rotation, whereas women may have favored verbal strategies. These findings indicate a tendency toward different strategy preferences rather than fixed sex-specific mechanisms. However, given the modest sample size, these findings should be interpreted cautiously and warrant replication in larger cohorts. Nonetheless, we conclude that when task demands increase, men may compensate by engaging mental object rotation.

**Supplementary Information:**

The online version contains supplementary material available at 10.1186/s13293-026-00880-2.

## Abstract (plain language summary)

We use touch every day to recognize objects and remember where they are. This kind of memory involves understanding both what an object is and where it is located. Remembering objects by touch uses skills that are also important for finding our way around spaces. Past research has shown that women and men often use different approaches when doing tasks that involve these skills. In this study, we looked at how people remember objects they explored using only touch. We found that women and men tended to use different strategies. Men were more likely to focus on where objects were located, while women were more likely to use words or labels to remember them. These results suggest that remembering objects by touch may depend partly on verbal ability. On average, women tend to do better on verbal tasks, while men may rely more on spatial information. The differences we observed were similar to those seen in other studies of spatial memory, showing that people may use different strengths when remembering objects through touch.

**Supplementary Information:**

The online version contains supplementary material available at 10.1186/s13293-026-00880-2.

## Highlights

- Sex-related tendencies influence strategy use during haptic object memory
- Men tended to preferentially engage spatial strategies, particularly when verbal/analytical strategies were less dominant, whereas women tended to engage verbal/analytical strategies
- Both spatial and verbal strategies contributed to haptic object recognition, with individual and sex-related differences in their relative use

**Supplementary Information:**

The online version contains supplementary material available at 10.1186/s13293-026-00880-2.

## Introduction

Everyday tasks often involve touch, such as finding a key in a handbag or pocket. Haptic object memory refers to the ability to perceive and identify objects through touch in the absence of other sensory input, such as vision or hearing [1–3]. Research has commonly assessed haptic recognition using stereognosis tests [4]. Haptic information is transmitted via the thalamus and the dorsal column–medial lemniscal pathway to the somatosensory and frontal cortices [5–8]. Microgeometric properties (e.g., texture) appear to be primarily processed in parietal regions, whereas macrogeometric features (e.g., object shape) may engage frontal cortical areas [7, 9]. Short-term maintenance likely involves non-verbal working memory mechanisms [10], while longer-term storage appears to rely on temporal lobe structures, particularly the hippocampus [11, 12]. Impairments in haptic memory have been observed in neurological and neurodevelopmental conditions, including probable Alzheimer’s disease [13], and may occur during both deliberate and passive exploration [14, 15]. Haptic processing shares representational features with vision, suggesting potential cross-modal mechanisms in object perception [14, 15].

Research on blind individuals—both congenitally and adventitiously blind—as well as blindfolded sighted participants, indicates that haptic exploration and spatial encoding can be important for memory performance [16–18]. Sighted individuals, even when blindfolded, typically rely on structured spatial processing strategies, such as sequential scanning and systematic mental transformation procedures that are rooted in visual experience. In contrast, blind individuals often develop compensatory strategies that integrate spatial and verbal encoding to support object representation and memory. These findings suggest that strategy selection—whether predominantly spatial or spatial-verbal—shapes haptic memory performance even in the absence of visual input.

### Cognitive style versus task-specific strategies

Strategy selection should be distinguished from cognitive style. Cognitive style reflects habitual processing preferences, such as holistic versus analytic approaches [19, 20], whereas strategies are flexible, goal-directed approaches used to accomplish specific tasks, such as mental rotation or verbal encoding [21, 22]. In haptic object memory, mental rotation exemplifies a spatial strategy, and reliance on verbal memory represents a verbal/analytical strategy. While these strategies may resemble aspects of cognitive or learning styles, they are distinct: learning styles reflect intrinsic preferences that do not necessarily optimize performance, whereas strategies are elective methods chosen to achieve task-specific goals. In this study, strategies refer to the approaches participants used to encode and retrieve haptic object information. The focus is on task-specific methods rather than overarching learning styles, with the present study examining sex differences in strategy selection during haptic object perception.

### Sex-dependent strategy use

Empirical evidence suggests probabilistic sex-related tendencies in cognition. Women often outperform men in verbal episodic memory tasks, whereas men may excel in spatial tasks, including mental rotation [23–28]. These tendencies could influence strategy selection—verbal/analytical or spatial/holistic—without implying fixed abilities. In haptic object memory, individuals might apply verbal strategies by labeling or semantically encoding objects, while spatial strategies could involve mentally aligning objects or integrating spatial configurations. Prior performance on verbal and spatial tasks may serve as markers of the tendency to engage corresponding strategies, though these measures do not guarantee equivalence with haptic memory performance.

### Gap and scientific rationale

Despite consistent evidence for sex differences in strategy use in verbal and spatial tasks, it remains unclear how these tendencies influence haptic object memory. Haptic memory relies on cross-modal representations that integrate tactile, spatial, and verbal information [29–32], and haptic exploration places substantial demands on spatial processing due to the sequential acquisition and integration of object features. Based on prior findings from spatial domains, we predicted a directional pattern: haptic object memory performance would be more strongly associated with spatial abilities in men and with verbal abilities in women. Furthermore, these associations were expected to be particularly evident during memory phases involving haptic encoding and retrieval. Demonstrating such effects would suggest that sex-related strategy tendencies extend to a sensory-specific domain and may be especially pronounced when spatially demanding haptic processing is required. These predictions are tested using an individual-differences approach, as outlined below.

Individual differences in haptic object memory may also be influenced by experiential factors that shape working memory and strategy selection. For example, prior gaming experience has been associated with enhanced spatial processing and mental rotation performance [33], while educational background can relate to verbal and analytical abilities. Including these variables allows us to account for secondary influences on strategy use and haptic memory performance without making them primary predictors of task outcomes.

### Study objectives and hypotheses

The present study examines how mental rotation (spatial strategy) and verbal memory (verbal/analytical strategy) may contribute to haptic object memory in men and women. We hypothesize that individual differences in verbal and spatial ability could predict the tendency to engage corresponding strategies, with probabilistic sex-related tendencies influencing strategy selection under task-specific conditions. Encoding may involve both sequential feature analysis and spatial integration, with verbal strategies engaged during labeling or semantic encoding, and spatial strategies recruited when aligning or integrating objects. By examining performance across encoding and recognition phases, the study aims to provide preliminary evidence regarding how individual differences and sex-related tendencies may influence strategy selection in haptic object memory. Importantly, this study uses an individual-differences approach rather than attempting to experimentally dissociate verbal versus spatial working memory, focusing on naturalistic strategy use during a newly developed haptic task.

## Methods

### Participants

Participants were recruited through advertisements on social media and on the premises of Ulm University at Bezirkskrankenhaus Günzburg. To minimize age-related variability in cognitive performance and to ensure a relatively homogeneous healthy sample, participation was restricted to early adulthood (≤ 36 years) [34–36]. Eligibility was assessed during recruitment using self-report screening. Participants were asked about current or past psychiatric or neurological disorders and relevant medical history, including prior diagnoses and treatment. A priori exclusion criteria were limited to incomplete data, failure to comply with task instructions, and self-reported psychiatric or neurological conditions. No additional exclusions were applied post hoc.

The final sample comprised 45 healthy young adults (18 male, 27 female; mean age = 26.8 years, *SD* = 4.8). Female participants were slightly younger than male participants (females: *M* = 26.3, *SD* = 4.6; males: *M* = 27.6, *SD* = 5.0). Educational attainment was evenly distributed, with 23 participants reporting no academic degree (eleven males, 12 females) and 22 reporting an academic degree (seven males, 15 females). Educational level was not included as a primary fixed effect in the main analyses but was examined in supplementary and exploratory analyses (see Supplementary Results). Educational level was included as a secondary factor, given its potential influence on verbal and analytical abilities that may affect strategy selection and haptic memory performance. Sex was defined as biological sex assigned at birth. Information on gender identity was not collected, as the present study focused on biological factors relevant to neuropsychological performance. Instead, sex was treated as a grouping variable reflecting biological classification at birth and was examined in relation to individual differences in cognitive strategy use, rather than as a deterministic explanatory factor.

The study was approved by the local ethics committee and conducted in accordance with the Declaration of Helsinki. Although the sample consisted of healthy young adults, the study was embedded in a clinical research framework. The ethics application specified the theoretical background, experimental procedures, assessed cognitive domains, and expected directional tendencies. Recruitment continued until this sample size was reached, with no interim analyses. Formal preregistration was not implemented. However, key design decisions—including outcome measures and exclusion criteria—were defined prior to data collection. Multivariate outliers were identified using Mahalanobis distance with a predefined chi-square threshold (α = 0.001). Participant exclusions were based on compliance and attentional criteria rather than post hoc statistical considerations. False discovery rate correction was applied across related analyses, and findings are explicitly identified as exploratory.

### Standardized tests

#### Questionnaire on gaming experience

Following the rationale described in the Introduction, gaming experience was assessed as a proxy for visuospatial exposure, which may influence strategy use and performance in haptic object memory tasks [33]. The participants were asked to rate the frequency of playing video games on a 5-point Likert scale (“How often do you play video games?” with 1 = never, 2 = seldom, 3 = sometimes, 4 = often, and 5 = very often). Gaming frequency was included as a proxy measure of visuospatial experience, given prior evidence linking video game exposure to mental rotation and spatial performance.

#### Measure of tactile acuity

All participants were asked about their handedness and were screened for tactile acuity using a two-point discrimination test (AFH Two-Point Discriminator Uno, AFH Webshop^®^, Germany). Tactile acuity was assessed at the fingertips using a standard procedure [37]. The examiner placed the device on the right thumb with a distance of 5 mm between the two points. Participants were asked to report whether they perceived one or two points. If a participant reported perceiving one point, the examiner continued with the next wider distance; if two points were reported, the examiner continued with the next smaller distance. The distance between the two points ranged from 2 mm to 12 mm. This procedure was repeated for all fingers. Values between 2 and 5 mm were interpreted as indicating normal tactile acuity [38]. All participants fell within the normal range (*M* = 2.88, *SD* = 0.56, *range* = 2–4.5). The test duration was approximately 5 min.

#### Mental rotation task

The mental rotation task was administered as an index of individual differences in spatial processing ability relevant to spatial strategy use. Mental rotation performance was not assumed to directly reflect haptic object memory performance, but rather to capture participants’ propensity to engage spatial imagery and object transformation strategies under task demands. The task was administered using Eprime (version 2.0). The stimuli were selected from an established stimulus library [39] and consisted of 16 standardized three-dimensional cube figures. These figures were rotated in 13 incremental steps around three main axes from 0° to 360°, and mirrored versions were also available. From this set, we selected 36 figures to achieve adequate reliability. Following the recommendations of the stimulus developers, we chose figures rotated around the y-axis, as this type of rotation creates a stronger sense of depth. The developers also suggested using three levels of rotation to represent increasing task difficulty. To balance difficulty, we selected three figures at each of six rotation angles, along with their corresponding mirror images: 15° and 45° (easy), 75° and 105° (medium), and 165° and 180° (hard). This resulted in a 2 (match: rotated vs. mirrored) × 3 (repeated items) × 6 (rotation angle) design, yielding a total of 36 trials per participant. Stimuli were displayed in the center of the screen and resized to an average of 700 × 300 pixels, occupying approximately 25% of a 24-inch LCD monitor. Each trial began with a fixation cross displayed for 3 s. If no response was made within 15 s, the trial ended and the task moved on to the next item. Response times were recorded for each trial. Participants were seated in front of a computer screen and viewed pairs of figures. On some trials, the figures differed by rotation around the y-axis, and on others they were mirror images. All trials were presented in random order. To encourage a consistent spatial reference frame, instructions emphasized the main axis of rotation. Participants were asked to decide whether each pair of figures was rotated or mirrored. Responses were made using the keypad (“1” = rotated, “2” = mirrored). Overall, the task required participants to judge whether pairs of three-dimensional figures were rotated or mirrored across increasing levels of angular difference, providing measures of both accuracy and response time.

#### California verbal learning test

The California Verbal Learning Test (CVLT) by Niemann 2008 [40] was administered to assess individual differences in verbal learning and memory relevant to verbal/analytical strategy use. Although episodic list learning differs from haptic object encoding in stimulus structure and retrieval demands, CVLT performance was used as an indirect indicator of participants’ tendency to rely on verbal strategies such as naming, labeling, and semantic organization. CVLT scores were therefore interpreted as reflecting strategy-relevant verbal learning capacity rather than direct analogues of haptic memory performance. The test provides scores for the learning trials in learning list A (L1 – L5), the learning trials in learning list B (LB), immediate free recall (VFWI), immediate cued recall (WAI), delayed free recall (VFWII) delayed cued recall (WAII), recognition hits and false alarms. The test was administered using standard procedures described in the test manual [40]. The participant is instructed to recall a list of 16 words. The word list is grouped in four categories. Standard version 1 was used in the present study (“Cucumber”). The learning trials are repeated five time for the first word list, and one time for a second interference list. The participant is instructed to immediately recall the first world list free and given the category as a cue. Recall was repeated after a 20 min delay. In recognition, the participant is given yes-no decisions whether a word had been presented in the first list.

### Experimental procedures

#### Haptic object memory

The haptic object memory task was designed to allow flexible, naturalistic strategy use during encoding and retrieval, rather than to constrain participants to a specific encoding strategy. The haptic object memory task comprised a set of ten objects with high everyday utility. Everyday utility was operationalized based on object familiarity, with screw, pen, key, coin, and doorstop categorized as high familiarity, and pipe, gemstone, pyramid stone, grinding disc, and wooden ring as low familiarity. For each target object, four semantically identical distractor objects differed from the target in at least one perceptual dimension (weight, height, or size). The degree of discrepancy between targets and distractors was counterbalanced according to perceptual similarity (0 = target item; ±1 = perceptually similar; ±2 = perceptually dissimilar).

*Encoding Phase*. During encoding, participants were seated face-to-face with the examiner and instructed to close their eyes while each object was placed in their hands. No restrictions were imposed on handedness, and participants were allowed to explore the objects freely using one or both hands. Exploration time was self-paced. Participants were then asked to name each object. If participants were unable to correctly name an object or used paraphrases, a single semantic cue describing the object’s functional use was provided (e.g., “This object is used for doors” for the doorstop). If participants remained unable to name the object after the cue, the examiner provided the correct name. This procedure ensured that all objects were verbally identified during encoding. Allowing free exploration and verbal identification was intended to permit participants to spontaneously engage spatial, verbal, or combined strategies, consistent with the study’s individual-differences approach.

*Retention Interval*. Following encoding, participants completed a 5-minute retention interval, during which tactile acuity was assessed using a two-point discrimination test. This intervening task was unrelated to object identity and was included to prevent active rehearsal of the encoded objects.

**Fig. 1:**
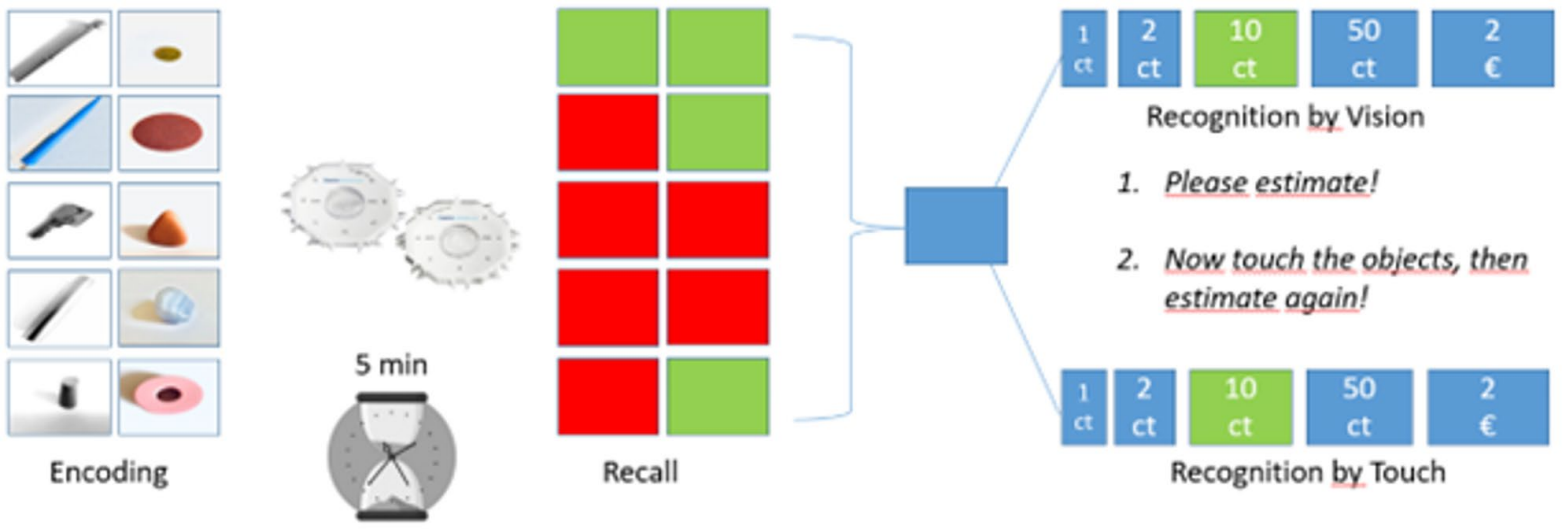
*Haptic Object Memory Task Procedure. Note*. Participants first encoded ten objects through haptic exploration (left panel). After a 5-minute delay, recall was assessed (middle panel) by reporting each object; green squares indicate correct recall and red squares indicate incorrect recall. Recognition was subsequently tested using vision and touch (right panel). Participants estimated the value of each object visually (“Recognition by Vision”) and then manually (“Recognition by Touch”). Color coding represents accuracy, and monetary labels indicate object value (ct = cents, € = euro)

*Retrieval Phase*. After the retention interval, participants first completed a free recall task, in which they were asked to verbally recall as many previously explored objects as possible. When participants were unable to recall an object, the same semantic cue used during encoding was provided once. Following free recall, participants completed a recognition task. For each target object that had been named during encoding, the target and four semantically identical but perceptually different distractor objects were presented simultaneously. Distractors varied systematically in weight, height, or size and were arranged in ascending order along the manipulated dimension. The position of the target object relative to the center was counterbalanced across trials. Participants were first asked to identify the previously explored object based on visual inspection. After making a selection, participants were instructed to touch all objects and confirm or revise their choice based on haptic information (see Fig. 1).

#### Scoring

For encoding and recall, participants were given 2 points if they scored correctly without help and 1 point if needed a cue; zero points were given if they failed completely on an item. Verbal assistance was treated as an integral component of the encoding process and explicitly modeled in the scoring system, rather than as uncontrolled variance. The responses in the two recognition trials were dichotomized into two separate accuracy scores for assessing the objects by vision versus by touch. Scores for all objects were added to calculate a summation score. Encoding (2 = encoded without help, 1 = encoded with help, 0 = forgotten), recall (2 = recalled without help, 1 = recalled with help, 0 = forgotten), recognition by touch and by vision (1 = recognized correctly, 0 = false estimate) in haptic object memory. Choosing the target item was scored as “1”. Choosing a distractor item was scored as “0”. Scoring procedures were designed to capture performance outcomes while preserving variability related to strategy use, rather than to penalize reliance on verbal assistance.

#### Task administration

All procedures were performed by an instructor trained in Good Clinical Practice and certified according to the standardized test’s administration requirements. The ethics committee of the Medical Faculty of the University of Ulm defined clear conditions for participant involvement. To investigate haptic object memory within the context of standardized neuropsychological testing, the order of tasks was held constant across participants. Participants first provided consent following ethical approval and then completed an assessment of demographic factors. Next, the CVLT (CVLT L1–L5 to CVLT VFWI) was administered, followed by haptic object memory encoding and measurement of tactile acuity. Haptic object memory recall was then assessed, and haptic object recognition was evaluated both visually and by touch. Participants subsequently completed a surrogate measure of online gaming frequency and were then administered the final part of CVLT (VFWII, recognition hits and false alarms). Afterwards, participants performed the mental rotation task. Finally, they were debriefed and thanked for their participation. Task order was held constant to preserve standardization across participants and to align with the administration requirements of standardized neuropsychological tests.

### Study design

The primary analytical focus was on how individual differences in verbal learning and spatial ability relate to performance across different phases of haptic object memory, and whether these relationships differ probabilistically by sex. Strategy use was not experimentally manipulated but inferred indirectly via individual differences in verbal learning (CVLT) and spatial ability (mental rotation), consistent with an individual-differences approach. Dependent variables were measured across tasks, with repeated measurements leveled on the item variable of the object memory task:

- Mental rotation task: accuracy (percentage of correct responses) and response time (milliseconds).
- CVLT: learning sum (sum of words learned during trials L1–L5), recall (sum of words recalled during trials VFW-I and VFW-II), recognition hits (sum of words correctly remembered) and false alarms (sum of words falsely remembered).
- Haptic object memory task: encoding (2 = encoded without help, 1 = encoded with help, 0 = forgotten), recall (2 = recalled without help, 1 = recalled with help, 0 = forgotten), and recognition by touch and by vision (1 = correctly recognized, 0 = incorrect).

## Results

Data were collected, organized, and analyzed using R (R Core Team, 2022). The dataset was structured to account for repeated measurements at the level of object items in the haptic memory task, with ten measurement points per participant corresponding to the object items.

To analyze performance outcomes, we fitted linear mixed-effects models (LMEs) using the “lmer” function from the R package lme4. LMEs were chosen to account for repeated measurements and to maintain a unified statistical framework across outcomes. LMEs included object items as random intercepts to control for repeated measures. Initial models included random intercepts for participants. However, these models resulted in singular fits indicating near-zero variance at the participant level. Therefore, the participant-level random effect was removed from the final models to avoid overparameterization while retaining item-level random intercepts to account for repeated measurements across objects. Each outcome variable (e.g., verbal memory learning sum, recognition hits, mental rotation accuracy) was analyzed in a separate univariate model. Therefore, only the residuals of each model are assumed to be approximately normally distributed; multivariate normality is not required. Residuals were visually inspected using Q-Q plots and showed no substantial deviations from normality. LMEs are robust to moderate violations of residual normality, supporting the validity of the analyses. Outliers were identified a priori using Mahalanobis distance. The critical χ² value for *p* < .001 (*df* = 4) was 18.47, and observed distances ranged from 0.49 to 39.10. A small number of participants exceeded this threshold; however, all participants were retained in the final analyses to avoid selective post hoc exclusions and ensure analytical consistency. Sensitivity analyses excluding extreme values yielded comparable results. Because multiple tests were conducted, *p*-values were corrected using the Benjamini-Hochberg FDR procedure to control for false discovery rate within each family of related outcome measures. All participants aged 36 years or younger were included. Educational level, gaming experience, and object familiarity were modeled as random effects to account for their influence without overparameterizing fixed effects. These variables were modeled as random intercept terms to absorb variance attributable to secondary experience-related factors, rather than to estimate population-level fixed effects. The dataset and all analysis scripts are publicly available on OSF (https://osf.io/mnuj5/overview), allowing full reproducibility of the reported results.

### Performance analyses

#### Verbal memory

LMEs were fitted separately for each verbal memory outcome (learning sum L1–L5, VFWI, VFWII, false alarms, and recognition hits) with sex (female vs. male) as a fixed effect and random intercepts for object items. Because multiple tests were conducted, p-values were corrected using the Benjamini-Hochberg FDR procedure. After correction, sex differences remained significant for learning sum trials L1 (FDR *p* < .001), L2 (FDR *p* = .002), L3 (FDR *p* < .001), L4 (FDR *p* < .001), L5 (FDR *p* = .002), VFWI (FDR *p* < .001), VFWII (FDR *p* < .001), indicating superior verbal learning and memory in female participants. No significant sex difference was observed for recognition hits (FDR *p* = .075) and false alarms (FDR *p* = .148). Across all significant outcomes, the effects indicated superior verbal learning and memory performance in female compared with male participants, reflected in higher learning scores, better delayed recall, whereas no reliable sex difference was observed for more recognition hits and false alarm rates. Descriptive statistics are reported in Table 1. Full model estimates are provided in Supplementary Table S1.

Including educational level, gaming experience, and object familiarity as additional random effects slightly attenuated sex differences in early learning (L1–L2), while later learning trials and recognition remained robust. Overall, these results indicate that sex differences favoring females were largely intact after accounting for secondary factors.

**Table 1:** Descriptive Statistics for Cognitive Performance by Sex

Outcome	Female (*n* = 27), mean (SD)	Male (*n* = 18), mean (SD)
**California verbal learning test (CVLT)**		
Learning Sum L1 (words)	9.0 (1.9)	8.1 (2.2)
Learning Sum L2 (words)	12.1 (2.1)	11.4 (2.6)
Learning Sum L3 (words)	13.6 (1.9)	12.8 (1.8)
Learning Sum L4 (words)	14.4 (1.3)	13.7 (1.8)
Learning Sum L5 (words)	15.1 (1.2)	14.7 (1.5)
Immediate Free Recall VFWI (words)	13.7 (1.9)	12.9 (2.0)
Delayed Free Recall VFWII (words)	14.3 (1.6)	12.8 (2.1)
Recognition hits (words)	15.6 (0.7)	15.7 (0.7)
Recognition false alarms (words)	0.1 (0.3)	0.2 (0.5)
**Haptic object memory**		
Encoding (a.u.) Recall (a.u.) Recognition by Vision (a.u.)	1.7 (0.5) 1.7 (0.6) 0.5 (0.5)	1.7 (0.5) 1.7 (0.6) 0.5 (0.5)
Recognition by Touch (a.u.)	0.5 (0.5)	0.5 (0.5)
**Mental rotation**		
Accuracy (%)	67.6 (15.8)	73.9 (10.9)
Response Time (ms)	5618 (1865)	6963 (1185)

All values are reported as mean (SD). CVLT measures include total words recalled across learning trials 1–5 (L1–L5), immediate free recall (VFWI), delayed free recall (VFWII), recognition hits, and false alarms. CVLT scores reflect the sum of words correctly recalled during the verbal memory task. Haptic object memory scores are unitless and reported in arbitrary units (a.u.); only relative differences are meaningful, with encoding scored as 2 = encoded without help, 1 = encoded with cue, 0 = not encoded, recall scored as 2 = recalled without help, 1 = recalled with cue, 0 = not recalled, and recognition by vision or touch scored as 1 = correct, 0 = incorrect. Mental rotation accuracy is expressed as proportion correct, and response times are reported in milliseconds. CVLT = California Verbal Learning Test

#### Mental rotation

LMEs for mental rotation accuracy and response time revealed significant sex differences, with males showing higher accuracy and faster responses. These effects remained robust after accounting for educational level, gaming experience, and object familiarity, and FDR correction confirmed that all effects were highly significant (FDR *p*s < .002). These results suggest reliable sex-related differences in mental rotation performance, reflected in both accuracy and processing speed. Complete fixed-effect estimates and model statistics are reported in Supplementary Table S1.

#### Haptic object memory

The number of semantic cues required during object naming at encoding was recorded for each participant. To assess potential sex differences in reliance on verbal or semantic assistance, we calculated the proportion of objects requiring a cue for each participant and compared males and females. Male participants required cues for 21.1% ± 9.0 of objects, and female participants required cues for 22.2% ± 9.3; this difference was not significant (Wilcoxon rank-sum test, *W* = 223, *p* = .633, indicating identical rank distributions).

LMEs for haptic memory outcomes—encoding, recall, recognition by touch, and recognition by vision—revealed no significant sex differences. FDR-corrected p-values were .871 for encoding, .871 for recall, .871 for recognition by touch, and .871 for recognition by vision, confirming comparable performance between male and female participants across all phases of the haptic memory task. These effects remained robust after accounting for educational level, gaming experience, and object familiarity (*p*s > 0.634). Full model estimates are provided in Supplementary Table S1.

Handedness, as described in the Methods section, was missing for four participants. Of those with reported handedness, 36.6% were right-handed males, 58.5% right-handed females, 2.4% left-handed males, and 2.4% left-handed females. Handedness did not differentially affect haptic memory performance, as models including a sex × handedness interaction did not improve fit for encoding (χ²(1) = 0.00, *p* = .951) or recognition by touch (χ²(1) = 1.05, *p* = .305).

### Strategy analyses

Prior research has demonstrated sex-specific patterns in verbal and spatial tasks, including differences in performance and neural activation [41, 42]. The present task requires participants to verbalize the stimuli while holding the items. It therefore appears appropriate to examine whether the learning sum, as an indicator of learning strategy, is associated with performance in the haptic object recognition task. Because the task involves the encoding of objects, analyzing learning strategies makes it possible to relate performance in haptic object memory to verbal memory measures that may reflect learning performance. In addition, as the haptic object memory task was newly developed, it is not yet clear which variable best captures learning performance in this test.

**Fig. 2:**
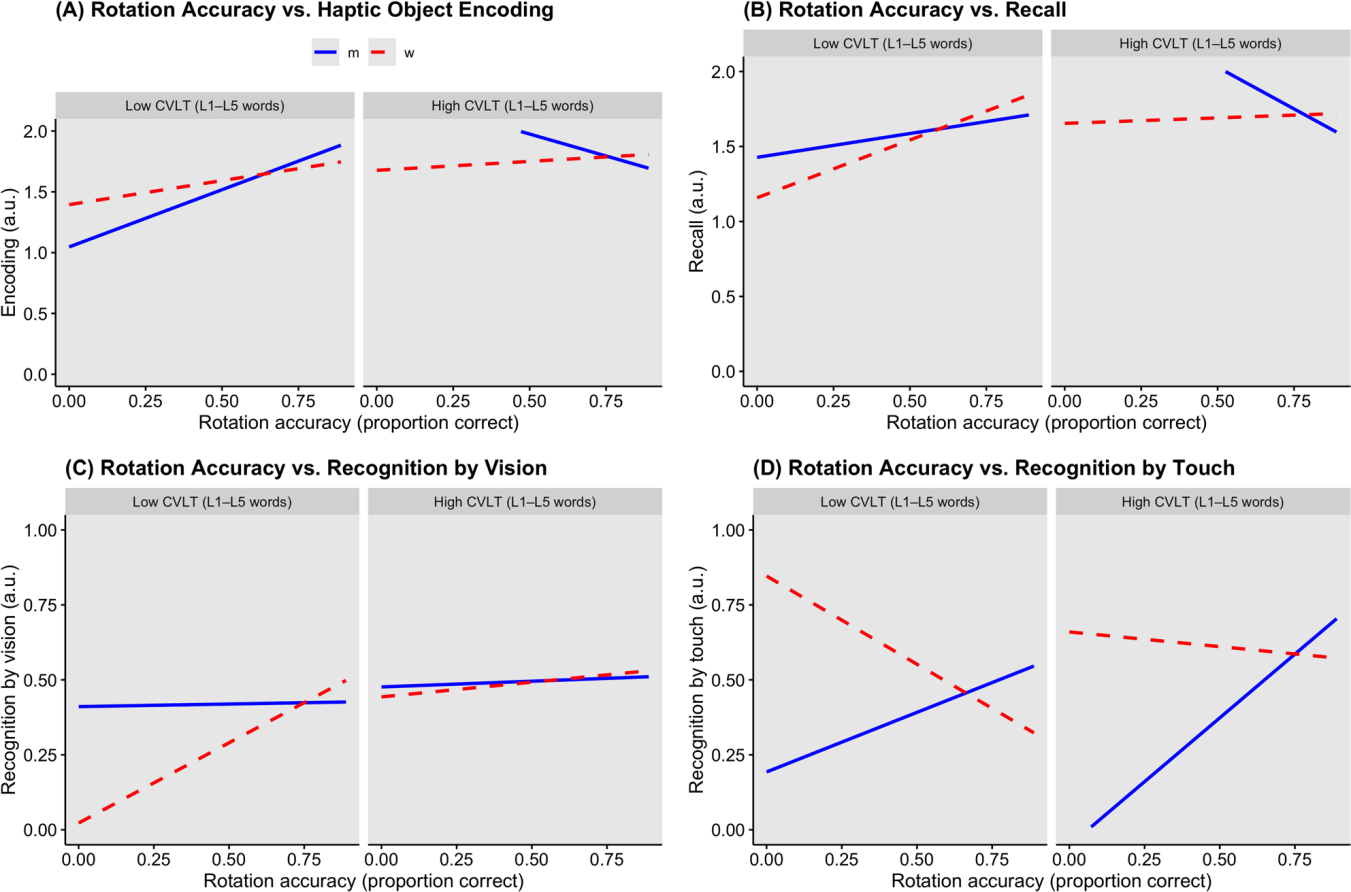
*Sex Differences in the Performance of the Haptic Object Memory Task Explained by the Performance in the Mental Rotation and the Verbal Memory Task* Associations between mental rotation accuracy and haptic object memory performance stratified by verbal memory ability. Panels show predicted values from linear mixed-effects models relating rotation accuracy (proportion correct) to (**A**) haptic object encoding, (**B**) object recall, (**C**) object recognition by vision, and (**D**) object recognition by touch, separately for females (red dashed lines) and males (blue solid lines). Models included fixed effects of sex, rotation accuracy, CVLT performance, and their interactions, with random intercepts for item. CVLT groups (low, medium, high) represent terciles of total words recalled across learning trials 1–5 (L1–L5). Haptic object memory outcomes are unitless and reported in arbitrary units (a.u.); encoding and recall are scored on a 0–2 scale, and recognition outcomes are scored as correct (1) or incorrect (0). Lines represent model-predicted means. A significant three-way interaction was observed for encoding (*χ*²(1) = 11.76, *p* = .008, FDR *p* = .033), indicating that the combination of verbal and spatial ability influenced encoding differently by sex. No significant interactions were observed for recall or recognition by touch or vision

To investigate cognitive strategies in haptic object memory, we fitted LMEs including sex, CVLT learning sum, and mental rotation accuracy as fixed effects. A significant three-way interaction was observed for encoding (χ²(1) = 11.76, *p* = .008, FDR *p* = .033), indicating that the combination of verbal learning and spatial ability influenced encoding differently by sex (see Fig. 2A). No significant interactions were observed for recall (see Fig. 2B) or recognition by vision (Fig. 2C) or touch (Fig. 2D). Full model coefficients for the interaction analyses are reported in Supplementary Table S2.

Follow-up analyses showed that, in men, higher mental rotation ability enhanced encoding when verbal memory was low (χ²(1) = 4.77, *p* = .029, FDR *p* = .044), consistent with a compensatory spatial strategy. In men with higher verbal memory and in women, encoding was less dependent on mental rotation ability. In recognition tasks, men’s performance was more strongly influenced by spatial ability, whereas women’s performance was primarily driven by verbal memory, highlighting sex-specific reliance on cognitive strategies during haptic object recognition. Follow-up analyses of sex-specific strategy effects in haptic object memory are reported in Supplementary Table S3.

### Exploratory analyses of experience-related factors

To further investigate the potential influence of experience-related variables, we conducted supplementary analyses in which educational level, gaming experience, and object familiarity were modeled as fixed effects and examined in interaction with sex. These analyses were not part of the primary inferential framework and were intended to assess whether these factors systematically moderated task performance. Among the five exploratory interactions tested, three remained significant after correction for multiple comparisons. Sex interacted with academic degree to predict verbal memory performance, with educational level showing a stronger association with learning outcomes in men than women. A similar sex-dependent association was observed for mental rotation accuracy, in which academic degree was more strongly related to performance in men. In addition, gaming experience moderated verbal memory performance in a sex-dependent manner, such that men and women differed in how prior gaming experience related to CVLT sum scores. In contrast, interactions between sex and gaming experience for mental rotation accuracy, as well as sex and object familiarity for encoding, did not survive correction for multiple comparisons. These findings extend the primary analyses by indicating that certain experience-related factors may influence cognitive performance in a sex-dependent manner. Because these analyses were exploratory and extend beyond the primary research questions, full statistical details and additional model results are reported in the Supplementary Results (Tables S1–S4) and should be interpreted as hypothesis-generating rather than confirmatory.

## Discussion

Haptic object memory is a complex cognitive process that likely integrates verbal encoding with spatial operations such as mental rotation, particularly when objects are explored in orientations that deviate from typical use. In the present study, we examined potential sex differences in verbal memory and mental rotation abilities and explored how these abilities relate to performance across distinct phases of haptic object memory, including encoding, recall, and recognition by touch or vision. Firstly, our results suggest that male and female participants may rely similarly on semantic assistance during encoding, indicating that observed sex differences in spatial processing are unlikely to be confounded by differential verbal mediation [41, 42]. Secondly, the findings are consistent with the possibility that haptic object memory performance is associated with sex-dependent patterns of verbal and spatial abilities, which appear to vary across memory phases. During encoding, linear mixed-effects analyses suggested that strategy use might depend on sex and individual ability profiles. In men with lower verbal memory, mental rotation ability showed a stronger association with encoding performance, consistent with the hypothesis that spatial processes could compensate when verbal resources are relatively limited. During later recognition phases, men appeared to continue relying on mental rotation for haptic recognition, whereas women tended to rely more on verbal memory. These patterns imply that cognitive strategies in haptic object memory may differ between sexes and across memory phases, although causal conclusions cannot be drawn.

Importantly, the haptic object memory task was designed to maximize ecological validity by allowing free object exploration and verbalization during encoding. While performance may involve working memory–related processes, the task does not experimentally isolate verbal versus spatial working memory subsystems. Dual- or double-interference paradigms, commonly used to dissociate these processes [43, 44], were not included. Consequently, inferences regarding working memory should be interpreted as suggestive rather than causal, and future studies could strengthen causal claims by incorporating interference manipulations.

### Sex-specific contributions of verbal and spatial strategies during haptic object memory encoding and recognition

Analysis of sex differences in the contributions of mental rotation and verbal memory across memory phases provided preliminary evidence that effects were most pronounced during encoding. Encoding in the present task likely reflects working memory–related processes, in line with prior research demonstrating sex differences in strategy use in other working memory–related tasks, such as spatial perception, navigation, and map learning. For example, fMRI data from navigation tasks suggest that men may preferentially engage hippocampal-dependent regions to support holistic–visual strategies, whereas women may engage parietal and prefrontal regions to support analytical–verbal strategies [25]. Consistent with this literature, our findings tentatively suggest that men may rely on mental rotation when verbal memory resources are limited, shifting toward verbal strategies when verbal memory is sufficient. This pattern could indicate that verbal memory represents a more efficient encoding strategy in this task, with mental rotation serving as a secondary or compensatory strategy.

In women, verbal memory performance was generally high during encoding, potentially reducing variability and attenuating its apparent association with encoding performance. The influence of verbal memory became more apparent during later recognition phases, where women consistently relied on verbal memory, while men relied more on mental rotation for haptic recognition. This pattern may reflect differences in cognitive processing demands across task phases, although neural interpretations remain speculative in the absence of neuroimaging data. Haptic recognition may place relatively greater demands on spatial processing, which could differentially relate to performance in men and women given known individual differences in spatial and verbal abilities. Empirical support for these neural interpretations would require neuroimaging studies.

Sex differences in strategy use appeared most prominent during memory phases involving touch and were less pronounced during recall or recognition by vision. Several factors may contribute to this pattern. Recognition tasks may impose lower executive demands than encoding, potentially making individual differences in performance more detectable. In contrast, cross-modal processing demands during visually guided recognition may mask sex differences during phases involving translation of haptic information into visual memory, which requires greater working memory resources. Moreover, haptic memory inherently emphasizes spatial features such as shape, orientation, and texture, making spatial imagery and mental rotation particularly relevant, whereas visual recognition may rely more on features such as color and surface detail.

### Sex differences in mental rotation and verbal memory: accuracy, speed, and strategy implications

Mental rotation performance showed sex differences when examined in isolation: accuracy was higher in men, whereas women responded faster. Mental rotation is a domain in which sex differences—most commonly favoring men—have been frequently documented, particularly in classic paper-and-pencil tasks involving complex three-dimensional stimuli [45–47]. At the same time, more recent computerized paradigms have reported reduced or absent sex differences, depending on task demands, response modalities, and time constraints [39, 48–50]. Differences in task format, verbal mediation, and stimulus characteristics—including whether objects are meaningful or meaningless—likely contribute to inconsistencies across studies [51–54].

Mental rotation tasks allow multiple solution strategies, including holistic rotation, piecemeal or feature-based comparison, and analytic approaches, which differ in speed and accuracy. Prior work suggests that men may be more likely to adopt holistic rotation strategies, whereas women may favor analytic or feature-based approaches, though substantial overlap exists between groups [25]. The observed dissociation—higher accuracy in men and faster responses in women—suggests that comparable task demands may be met via different cognitive strategies. These differences are relevant for interpreting sex-dependent strategy use in haptic object memory, although conclusions remain tentative.

Consistent with prior meta-analyses, women outperformed men in verbal learning across repetitions and in delayed recall [26, 27]. These effects were generally small and may be influenced by demographic factors such as education and age. Exploratory analyses indicated that educational attainment was more strongly associated with verbal memory performance in men than in women, suggesting that variability in educational background may contribute to individual differences in verbal learning performance and strategy use.

### Limitations & future directions

Several limitations should be noted. First, the study focused on early adulthood, a developmental period that remains relatively understudied in haptic object memory research. Second, reliance on manual object exploration may limit generalizability to increasingly digitalized contexts. Third, the modest sample size limits statistical power for detecting higher-order interactions and sex-stratified effects, though linear mixed-effects models partially mitigate this limitation. Fourth, mental rotation and verbal memory were used as indirect indicators of strategy use, as no self-report or process-level strategy measures were collected. Consequently, strategy use was inferred from correlational associations rather than assessed directly, limiting causal interpretation. Fifth, object retrieval followed a filled retention interval and involved semantic cueing, indicating that performance reflects a combination of short-term episodic and longer-term memory processes rather than pure working memory. Finally, the absence of verbal and spatial interference paradigms limits causal inference regarding specific working memory subsystems. Future studies incorporating interference tasks, explicit strategy assessments, larger samples, and neuroimaging approaches would strengthen understanding of the mechanisms supporting haptic object memory.

Given that the processes and mechanisms underlying haptic object memory are relatively understudied, the present findings primarily generate informed hypotheses. By demonstrating potential sex-dependent strategy use, the findings are consistent with the view that haptic object memory may involve flexible cognitive processes that vary across individuals and task demands. Mental rotation and verbal memory—both well-studied and sex-dependent abilities—appear to differentially support haptic object memory performance. Exploratory analyses suggest that experience-related factors such as gaming experience and educational background may also modulate these cognitive processes. Future research should directly investigate how experience shapes strategy selection.

### Conclusion

In sum, the present findings indicate that haptic object memory performance is associated with sex-dependent patterns of verbal and spatial abilities, with these associations varying across task phases and individual ability profiles. On average, men tend to recruit spatial strategies more when verbal memory is relatively low, whereas women tend to rely more on verbal strategies. These patterns are probabilistic rather than deterministic and should be interpreted in the context of task-specific demands and individual differences. By accounting for secondary factors such as prior experience and education, and by transparently discussing multiple limitations, the present study provides a framework for understanding how verbal and spatial strategies may contribute to haptic object memory. Future research integrating interference paradigms, explicit strategy assessments, larger samples, and neuroimaging approaches will be essential to further clarify the causal mechanisms and experiential influences underlying these sex-dependent patterns.

## Supplementary Information

Below is the link to the electronic supplementary material.

Supplementary Material 1

## Data Availability

The datasets generated and/or analysed during the current study are publicly available in the OSF repository: (https://osf.io/mnuj5).
