# Supplementary material for: Sex differences in the strategies of haptic object memory in early adulthood
Source: Biol Sex Differ. 2026 Mar 24;17:72. doi: 10.1186/s13293-026-00880-2 (PMC13063925; doi:10.1186/s13293-026-00880-2)
Supplement: Supplementary file 1 — Supplementary Material 1 [file 13293_2026_880_MOESM1_ESM.docx]

**Supplementary Results**

[Sex Differences in the Strategies of Haptic Object Memory in Early Adulthood]

[Lea Fröscher & Matthias W. Riepe]

SUPPLEMENTARY RESULTS

**Table S1**

*Primary Linear Mixed-Effects Models for Sex Differences in Cognitive Performance*

| Outcome | Dependent Variable | *Fixed Effect* (Female > Male) | Estimate ($\beta$) | SE | $\chi$^2^(*df*=1) | *p* value | FDR *p* value |
| --- | --- | --- | --- | --- | --- | --- | --- |
| CVLT | L1 | Sex | 0.91 | 0.19 | 21.48 | <.001 | <.001 |
|  | L2 | Sex | 0.70 | 0.22 | 10.01 | .002 | .002 |
|  | L3 | Sex | 0.81 | 0.18 | 20.53 | <.001 | <.001 |
|  | L4 | Sex | 0.74 | 0.15 | 23.93 | <.001 | <.001 |
|  | L5 | Sex | 0.41 | 0.13 | 10.24 | .001 | .002 |
|  | VFWI | Sex | 0.80 | 0.19 | 17.76 | <.001 | <.001 |
|  | VFWII | Sex | 1.46 | 0.17 | 66.08 | <.001 | <.001 |
|  | Recognition Hits | Sex | -0.13 | 0.07 | 3.37 | .066 | .075 |
|  | Recognition False Alarms | Sex | -0.06 | 0.04 | 2.09 | .148 | .148 |
|  |  |  |  |  |  |  |  |
| Mental Rotation | Accuracy | Sex | -0.06 | 0.01 | 21.40 | <.001 | <.001 |
|  | Response Time | Sex | -1344.5 | 156.3 | 68.52 | <.001 | <.001 |
|  |  |  |  |  |  |  |  |
| Haptic Object Memory | Encoding | Sex | -0.01 | 0.03 | 0.12 | .728 | .871 |
|  | Recall | Sex | 0.01 | 0.05 | 0.06 | .809 | .871 |
|  | Recognition by Vision | Sex | 0.01 | 0.05 | 0.03 | .871 | .871 |
|  | Recognition by Touch | Sex | 0.01 | 0.05 | 0.04 | .838 | .871 |

*Note*. Fixed-effect estimates from linear mixed-effects models examining sex differences in verbal memory, mental rotation, and haptic object memory performance. Models included random intercepts for object items. P-values were corrected within outcome families using the Benjamini–Hochberg false discovery rate (FDR) procedure.

^a^ CVLT refers to California Verbal Learning Test (Learning Sum L1 to L5, Immediate Free Recall VFWI, Delayed Free Recall VFWII, Recognition Hits and False Alarms).

**Table S2**

*Strategy Models: Interactions Between Sex, Verbal Learning, and Mental Rotation*

| Outcome | *Fixed Effect* | Estimate ($\beta$) | SE | $\chi$^2^(*df*=3) | *p* value | FDR *p* value |
| --- | --- | --- | --- | --- | --- | --- |
| Encoding | Sex × CVLT Learning Sum × Mental Rotation Accuracy | 0.09 | 0.04 | 11.76 | .008 | .033 |
| Recall | Sex × CVLT Learning Sum × Mental Rotation Accuracy | 0.05 | 0.08 | 3.36 | .340 | .679 |
| Recognition by Vision | Sex × CVLT Learning Sum × Mental Rotation Accuracy | -0.03 | 0.06 | 0.76 | .859 | .859 |
| Recognition By Touch | Sex × CVLT Learning Sum × Mental Rotation Accuracy | 0.00 | 0.06 | 1.18 | .757 | .859 |

*Note*. Fixed-effect estimates from linear mixed-effects regression models examining interactions between sex, verbal learning (CVLT learning sum), and mental rotation accuracy on haptic object memory performance. P-values were corrected using FDR within the strategy analysis family.

^a^Table S2 reports likelihood-ratio test statistics for three-way interaction effects only, as these constituted the primary inferential tests in the strategy analyses and formed the basis for the multiple-comparison correction. Main or two-way interaction effects were not subject to FDR correction. ^b^CVLT refers to California Verbal Learning Test (Learning Sum L1 to L5

**Table S3**

*Follow-up Analyses for Sex-Specific Cognitive Strategy Effects in Haptic Object Memory*

| Effect | $\chi$^2^ | df | *p* value | FDR *p* value |
| --- | --- | --- | --- | --- |
| (a) Mental rotation × CVLT L1–L5 in men | 9.14 | 1 | .003 | .015 |
| (b) Mental rotation × CVLT  L1–L5 in women | 0.33 | 1 | .565 | .565 |
| (c) Mental rotation effect in men with low verbal memory | 4.77 | 1 | .029 | .044 |
| (d) Mental rotation effect in men with high verbal memory | 2.60 | 1 | .107 | .128 |
| (e) Mental rotation effect on recognition by touch in men | 4.84 | 1 | .028 | .044 |
| (f) Mental rotation effect on recognition by touch in women | 4.79 | 1 | .029 | .044 |

*Note*. Likelihood-ratio tests compare models including the interaction/contrast of interest with reduced models excluding it. Random intercepts for object items were included. FDR correction was applied across the six effects within this follow-up family. CVLT = California Verbal Learning Test. Only effects relevant to the post hoc follow-up strategy analyses are shown; full model coefficients are available upon request.

**Table S4**

*Exploratory Analyses of Experience-Related Moderators*

| Outcome | Predictor | Modell Comparison | $\chi$^2^ | *df* | *p* value | FDR *p*  value |
| --- | --- | --- | --- | --- | --- | --- |
| Mental rotation accuracy | Sex × Gaming experience | Full vs. reduced LME | 6.46 | 3 | .091 | .114 |
| Mental rotation accuracy | Sex × Academic Degree | Full vs. reduced LME | 39.62 | 1 | <.001 | <.001 |
| CVLT Learning Sum | Sex × Gaming experience | Full vs. reduced LME | 122.75 | 3 | <.001 | <.001 |
| CVLT Learning Sum | Sex × Academic Degree | Full vs. reduced LME | 8.41 | 1 | .004 | .006 |
| Haptic object encoding | Object Familiarity | Full vs. reduced LME | 0.86 | 1 | .354 | .354 |

*Note*. LMEs = linear mixed-effects models. Full vs. reduced LME comparisons indicate whether including the specified predictor or interaction improved model fit relative to a model without it. χ² = likelihood ratio test statistic; df = degrees of freedom. p values are reported alongside FDR-corrected p values to account for multiple comparisons. Outcome variables include mental rotation accuracy, CVLT learning sum, and haptic object memory performance. Separate follow-up analyses were conducted for men and women where interactions with sex were significant. Object familiarity was examined as a predictor but did not significantly influence haptic memory performance.
